# Serum Adipsin Levels throughout Normal Pregnancy and Preeclampsia

**DOI:** 10.1038/srep20073

**Published:** 2016-02-01

**Authors:** Natalia E. Poveda, María F. Garcés, Carlos E. Ruiz-Linares, Diana Varón, Sergio Valderrama, Elizabeth Sanchez, Adriana Castiblanco-Cortes, Yessica Agudelo-Zapata, Héctor Fabio Sandoval-Alzate, Luis G. Leal, Edith Ángel-Müller, Ariel I. Ruíz-Parra, Angélica M. González-Clavijo, Carlos Diéguez, Rubén Nogueiras, Jorge E. Caminos

**Affiliations:** 1 Department of Physiology, School of Medicine, Universidad Nacional de Colombia, Bogotá, Colombia; 2 Department of Obstetrics and Gynecology, School of Medicine, Universidad Nacional de Colombia, Bogotá, Colombia; 3 Department of Physiology (CIMUS), School of Medicine-Instituto de Investigaciones Sanitarias (IDIS), University of Santiago de Compostela, Santiago de Compostela, Spain; 4 CIBER Fisiopatología de la Obesidad y Nutrición (CIBERobn), Spain

## Abstract

Adipsin is a protease produced at high levels by adipose tissue. It is involved in complement activation and metabolic control. The objective of this study was to determine the changes in adipsin levels during different stages of normal pregnancy, and its association with obstetric outcomes, such as preeclampsia. This nested case-control study in a longitudinal cohort included normal pregnant (n = 54) and preeclamptic (n = 18) women, both followed throughout pregnancy. Additionally, some of the normal pregnant women were followed up three months postpartum (n = 18). Healthy non-pregnant women were also studied during their menstrual cycle (n = 20). The results of this study show that in healthy non-pregnant women, adipsin levels did not change significantly during the menstrual cycle. In normal pregnant women, adipsin levels were lower (p < 0.01) when compared with non-pregnant healthy women, but these serum levels increased again during postpartum (p < 0.001). Adipsin levels were significantly elevated in preeclamptic women in late pregnancy (P < 0.01). A significant correlation was not found between leptin and adipsin during the three periods of gestation studied in healthy pregnant and preeclamptic women. Our results suggest that adipsin may be involved in pregnancy-associated metabolic changes. Moreover, the increase of adipsin levels towards late gestation in preeclamptic women could be related to the pathophysiology of this disease.

Adipsin (also known as Complement Factor D, C3 convertase activator) is a serine protease expressed and secreted at high levels by adipose tissue^1^. Adipsin cleaves Factor B (FB) when this is found coupled to C3b, giving rise to the C3bBb complex (C3 convertase) of the alternative pathway of the complement activation^2^. This complex in turn cleaves C3 into its active components C3a and C3b^2^. Additionally, in the circulatory system, the terminal arginine of C3a is cleaved by carboxypeptidase B (CbB), generating an acylation-stimulating protein (C3adesArg/ASP)^3^. Both C3a and ASP, interact with the receptor C5L2 to stimulate triglycerides (TG) synthesis in cultured adipocytes^3^,^4^.

During pregnancy, an increased activation of the complement system has been described^5^. Richani *et al.* showed that during normal gestation, the innate immune system is activated and plasma concentrations of C3a, C4a, and C5a rise^5^. Thus, it has been proposed that this elevation of complement components could offset the suppression of adaptive immunity during normal pregnancy. Furthermore, it has been shown that terminal complement complexes and protein S are deposited in both normal and preeclamptic placentas, with a greater amount deposited in the latter^6^. The complement also promotes innate immunity, especially acting in areas of active inflammation^7^. It has been shown that in human and murine activation, the third and fourth complement components (C3 and C4) are regulated by the bound membrane proteins Decay Accelerating Factor (DAF) and Membrane Cofactor Protein (MCP), whereas in rodents the presence of the protein Crry was demonstrated^8^. Additionally, Crry deficient mice (Crry^**−/−**^) showed embryonic lethality, as they were unable to suppress spontaneous complement activation and tissue inflammation in the decidua and trophectoderm areas of the placenta^7^.

The pathophysiology of preeclampsia probably involves maternal, fetal and placental factors^9^. Abnormalities in the development of the placenta vasculature during pregnancy may lead to hypoperfusion, placental hypoxia and ischemia. This leads to the release of angiogenic factors in the maternal circulation, altering endothelial function, causing systemic hypertension and other manifestations of disease^10^. However, the molecular basis for the deregulation of placenta remains unknown, and the role of angiogenic proteins in placental vascular development is under investigation. It has also been reported that metabolic factors are involved in the pathophysiology of preeclampsia, where the adipocyte plays an important role in terms of production of proinflammatory cytokines with multiple endocrine functions and roles in oxidative balance^11^. Furthermore, insulin resistance and hyperinsulinemia may contribute to the metabolic syndrome of pregnancy which is associated with oxidative stress and endothelial dysfunction^13^. Preeclamptic women had a significant reduction in the levels of high-density lipoprotein cholesterol (HDL-cholesterol) and increased levels of triglycerides and insulin levels, when compared to a group of healthy pregnant women^12^.

This relationship between complement activation and preeclampsia has been described for decades^13^,^14^,^15^. Measurement of complement activation products have demonstrated that complement activation is greater in preeclamptic pregnancies compared to normal pregnancies^16^. Derzsy *et al.* showed both C3a/C3 ratio and sC5b9 levels increased and levels of C3 decreased in preeclamptic patients when compared to normal pregnant women^17^. Several studies have used complement inhibitors for the treatment of preeclampsia with promising results^18^,^19^,^20^. Moreover, a relation was found between complement activation and adverse pregnancy outcomes such as intrauterine growth restriction (IUGR) and gestational diabetes^17^,^21^. Finally Wang *et al.* showed that adipsin levels were significantly elevated in the urine of patients with preeclampsia^22^.

Regarding the role of metabolic control, adipsin stimulates glucose transport and adipocyte triglyceride synthesis through an insulin-dependent mechanism^23^,^24^. Human studies have shown that adipsin levels are increased in obesity and type 2 diabetes, whereas exercise or weight loss decreases these levels^3^,^23^,^25^,^26^,^27^. Likewise, it has been found that different complement proteins are expressed in adipose tissue, including adipsin^28^. Recently, it has also been demonstrated that the adipsin and C3a pathway may be the result of a connection between the adipocyte and pancreatic β cell. Adipsin knockout mice showed significant changes in parameters related to glucose homeostasis when subjected to diet-induced obesity^29^. In this same study, C3AR1 receptor expression was demonstrated in pancreatic β cells, and it was confirmed *in vitro* that C3a but not C3, significantly induces insulin secretion in islet β cells in the presence of high concentrations of glucose^29^. In this sense, ASP, another downstream molecule in the complement pathway, in addition to its involvement in the local regulation of lipid formation in adipocytes, increases insulin secretion, a glucose dependent effect^30^. These studies demonstrated that some components of the complement have a direct effect on the pancreatic β cell, promoting the insulin release.

Since adipsin plays an important role in fat metabolism and glucose homeostasis, two aspects that are markedly altered during gestation and preeclampsia, this study aims to investigate maternal serum adipsin levels during the normal gestation and preeclamptic women.

## Results

Demographic data and biochemical characteristics of healthy non-pregnant, normal pregnant, and preeclamptic women can be seen in Supplementary Table 1, Tables 1 and 2, respectively. As expected, in the healthy non-pregnant women, progesterone levels were significantly elevated during the luteal phase when compared to the follicular phase of the menstrual cycle (Supplementary Table 1). The anthropometric and biochemical parameters of healthy non-pregnant women are within normal limits (Supplementary Table 1). In normal pregnancy, anthropometric and biochemical changes which are characteristics of gestation were observed over the progression of the three periods studied, such as those seen in BMI (Body Mass Index), HOMA (Homeostasis model assessment), serum triglycerides, total cholesterol, and HDL-cholesterol, among others (Table 1). In preeclamptic women, significant differences were observed in systolic blood pressure in early and middle pregnancy and in BMI in middle and late pregnancy when compared with the normal pregnant group (Supplementary Table 2). Furthermore, there was a very significant reduction of HDL-cholesterol levels in the group of preeclamptic women in comparison with the normal pregnancy group towards the end of gestation (Supplementary Table 2).

### Serum adipsin and leptin levels in healthy non-pregnant, normal and preeclamptic pregnant women

**Figure 1:**
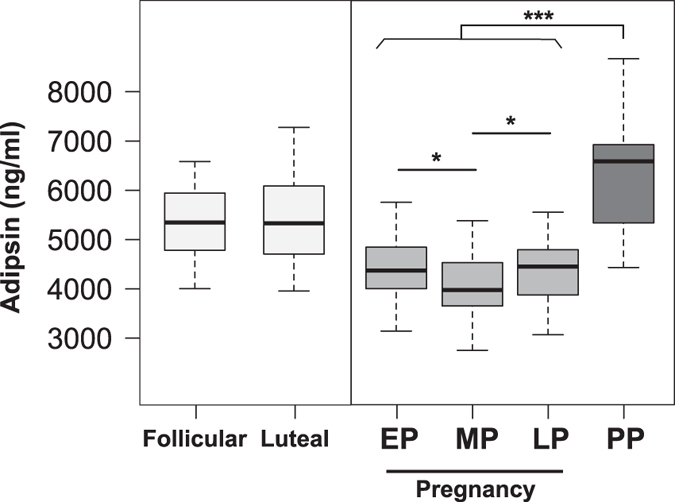
Serum adipsin levels across pregnancy compared with three months postpartum and non-pregnant controls during both the follicular and luteal phase. Box-and-whisker plot with median value, interquartile range, and lower and upper values. No significant differences were observed between the follicular and luteal phase. A significant decrease in serum adipsin levels was observed across normal pregnancy compared with healthy non-pregnant women and three months postpartum; also there was a significant decrease in adipsin levels in middle pregnancy compared with early pregnancy. EP = early pregnancy, MP = middle pregnancy, LP = late pregnancy, PP = postpartum. The statistical significance is indicated by *p < 0.05 and ***p < 0.001.

Serum adipsin levels did not show significant differences (p > 0.05) in the group of healthy non-pregnant women between the follicular and luteal phase of the menstrual cycle (Fig. 1 and Supplementary Table 1). On the other hand, adipsin serum levels were significantly lower (p < 0.01) in all the periods of the normal pregnant group in contrast to the serum levels of healthy non-pregnant women (Fig. 1). In addition, serum adipsin levels were significantly lower (p < 0.001) in the three periods of pregnancy when compared with serum adipsin levels of three months postpartum group (Fig. 1). In normal pregnant women, serum adipsin levels were significantly lower in the middle stage of pregnancy when compared to early pregnancy levels (p < 0.05) (Fig. 1, Table 1).

**Figure 2:**
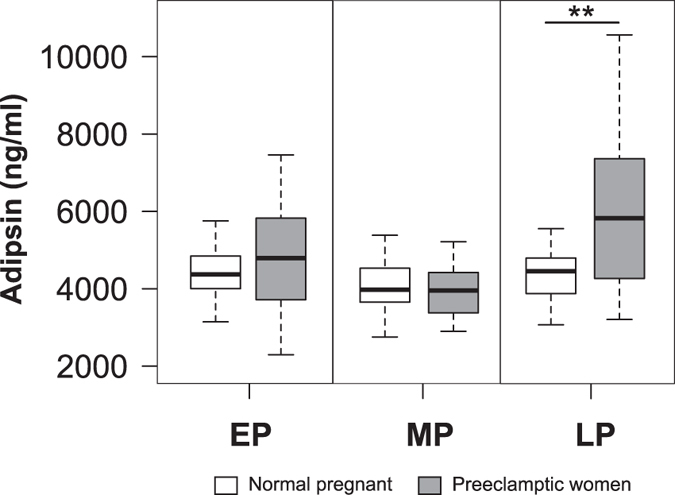
Serum adipsin levels in normal pregnant women compared with preeclamptic women across pregnancy. A significant increase of serum adipsin levels was observed in preeclamptic women in late pregnancy when compared with normal pregnant women. No significant differences were observed in the other periods analyzed. Open boxes are normal pregnant women and shaded boxes are pregnant women with preeclampsia; EP = early pregnancy, MP = middle pregnancy, LP = late pregnancy. The statistical significance is indicated by **p < 0.01.

**Figure 3:**
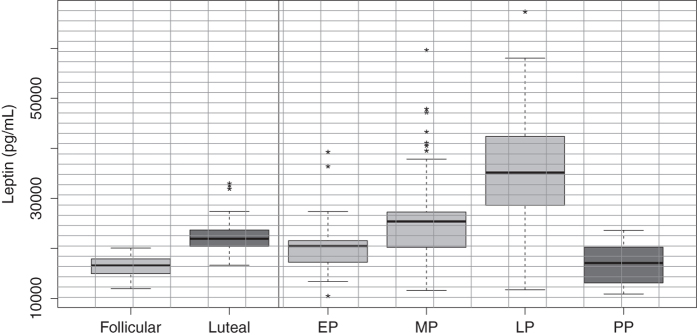
Serum leptin levels during pregnancy, three months postpartum and non-pregnant women during both the follicular and luteal phase. Box-and-whisker plot with median value, interquartile range, and lower and upper values. Significant differences were observed between the follicular and luteal phase. A significant increase in serum leptin levels was observed across normal pregnancy compared with healthy non-pregnant women and three months postpartum. EP = early pregnancy, MP = middle pregnancy, LP = late pregnancy, PP = postpartum. The statistical significance is indicated by *p < 0.05and ***p < 0.001.

When comparing serum adipsin levels throughout the three periods of gestation between normal pregnant women and preeclamptic women without features of severity, the levels differed only in late pregnancy, being significantly higher in preeclamptic women (P < 0.01). No differences were found between normal pregnancy and preeclamptic pregnant women in the early and middle stages of pregnancy (Fig. 2, Supplementary Table 2). Furthermore, leptin levels were significantly higher (p < 0.001) in the luteal phase (day 21–22) when compared to the follicular phase (day 4–5) of the menstrual cycle (Supplementary Table 1 and Fig. 3), which is consistent with previous studies^31^.

Additionally, it was observed that serum leptin levels were significantly lower in healthy non-pregnant women in the follicular phase (p < 00.1) when compared to healthy pregnant women in each period of gestation studied. Leptin levels were significantly increased (p < 0.001) with the advance of normal pregnancy. In turn, in each of the three trimesters of pregnancy, serum leptin levels were significantly higher when compared with postpartum levels (Table 1 and Fig. 3). Overall, our results are similar to previously reported studies^32^,^33^.

**Figure 4:**
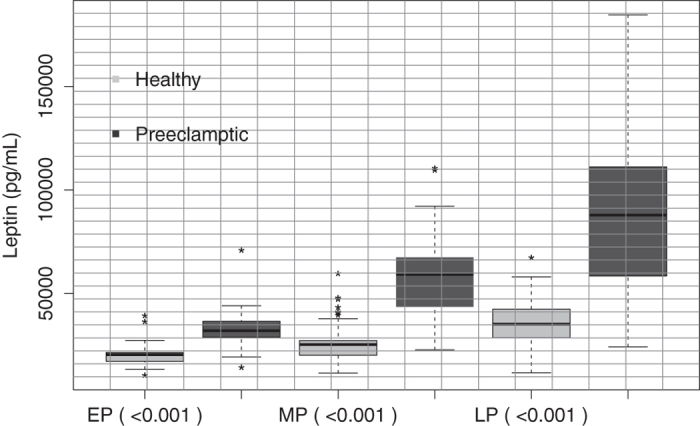
Serum leptin levels in normal pregnant women compared with preeclamptic women during pregnancy. A significant increase in serum adipsin levels was observed in preeclamptic women compared with normal pregnant women, in each gestational periods studied. Open boxes are normal pregnant women and shaded boxes are pregnant women with preeclampsia; EP = early pregnancy, MP = middle pregnancy, LP = late pregnancy. The statistical significance is indicated by **p < 0.01.

Finally, this study shows that the serum leptin levels in each of the three periods of gestation studied are significantly higher in preeclamptic women when compared with leptin levels in normal pregnant women (p < 0.001) (Supplementary Table 2 and Fig. 4). These results are similar to results previously described^34^,^35^,^36^

### Correlations between adipsin and leptin levels, anthropometric, clinical, and biochemical features in healthy non-pregnant women

**Figure 5:**
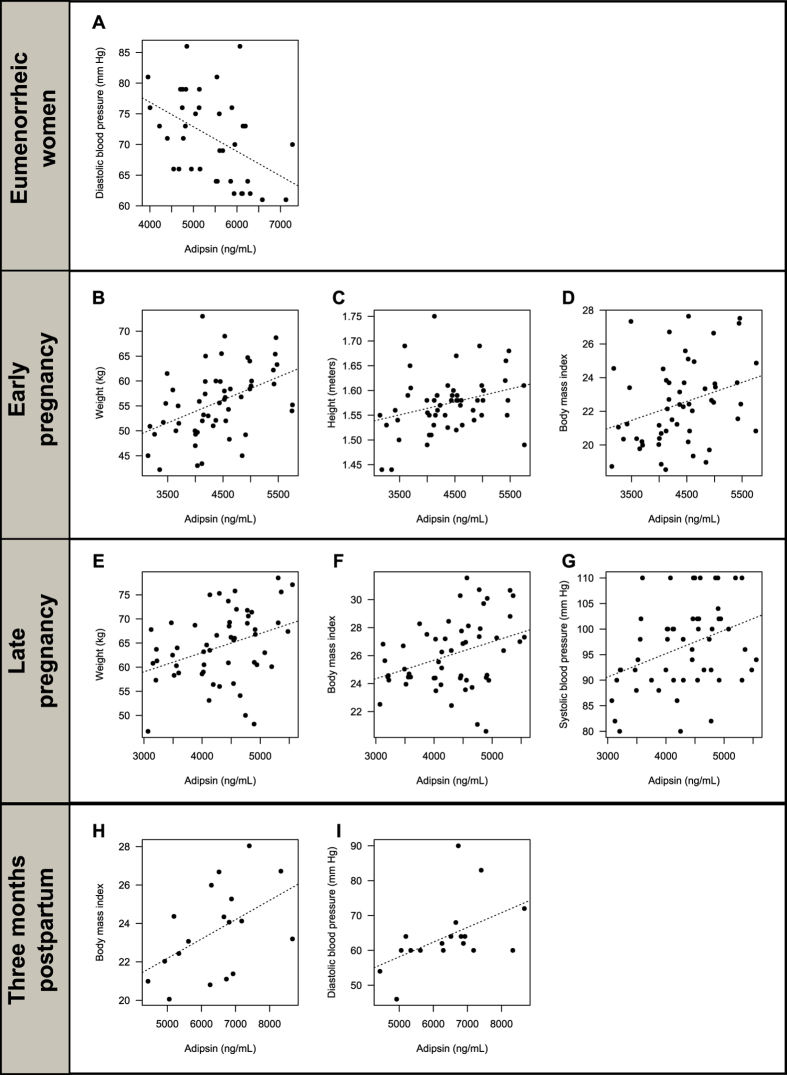
Scatterplots of anthropometric/biochemical variables correlated with adipsin in healthy non-pregnant (eumenorrheic) women. (**A**) Negative correlation between serum adipsin levels and diastolic BP in healthy non-pregnant women. (**B–D**) Positive correlations between adipsin levels and weight, height, and BMI in early pregnancy. (**E–G**) Positive correlations between adispin levels and weight, BMI, and systolic BP in late pregnancy. (**H,I**) Positive correlations between adipsin levels, BMI, and diastolic BP in women three months postpartum.

In the healthy non-pregnant women, a strong negative correlation between adipsin levels and diastolic blood pressure (BP) (r = −0.5 and p < 0.01) was observed (Fig. 5A, Supplementary Table 3). No significant correlation (Supplementary Table 3) was found between adipsin and leptin levels in follicular and luteal phase of the menstrual cycle.

### Correlations between adipsin and leptin levels and anthropometric, clinical, and biochemical features in normal pregnant women

In the group of normal pregnant women, positive correlations between serum adipsin levels and body weight (r = 0.49; p < 0.00) (Fig. 5B), height (r = 0.31; p = 0.02) (Fig. 5C), and the BMI (r = 0.34; p = 0.01) (Fig. 5D) were observed in early pregnancy (Supplementary Table 3). In late pregnancy, there was a positive correlation between serum adipsin levels and body weight (r = 0.38, p = 0.01) (Fig. 5E), BMI (r = 0.39; p = 0.01) (Fig. 5F), and systolic BP (r = 0.41; p < 0.00) (Fig. 5G, Supplementary Table 3). In the present study no significant correlation was found between leptin levels and adipsin in each of the trimesters analyzed (Supplementary Table 3).

### Correlations between adipsin levels and leptin, anthropometric, clinical, and biochemical features in preeclamptic women

In preeclamptic women, a strong positive correlation between serum adipsin levels and BMI (r = 0.87; p < 0.00) (Supplementary Fig. 1A) and HDL-cholesterol levels (r = 0.57; p = 0.04) (Supplementary Fig. 1B) was observed in early pregnancy. A strong negative correlation between the adipsin levels and triglycerides levels (r = −0.57; p = 0.04) (Supplementary Fig. 1C) was observed in early pregnancy (Supplementary Table 3). Also, in late pregnancy, strong negative correlations between the adipsin levels and systolic BP (p < 0.0 r = −0.77) (Supplementary Fig. 1D), glucose levels (r = −0.77; p < 0.00) (Supplementary Fig. 1E), total cholesterol (r = −0.76; p < 0.00) (Supplementary Fig. 1F), and LDL-cholesterol levels (r = −0.92; p < 0.00) (Supplementary Fig. 1G) were observed (Supplementary Table 3). Additionally, no significant correlation between the levels of adipsin and leptin was found in each of the three trimesters of gestation (Supplementary Table 3).

### Correlations between adipsin levels and leptin, anthropometric, clinical, and biochemical features in healthy postpartum women

In the group of healthy postpartum women, positive correlations between adipsin levels and BMI (r = 0.51; p = 0.03) (Fig. 5H) and diastolic BP (r = 0.55; p = 0.02) (Fig. 5I) were observed (Supplementary Table 3). Finally, no significant correlation between serum levels of adipsin and leptin found postpartum (Supplementary Table 3).

## Discussion

### Adipsin serum levels throughout pregnancy

The present study shows that serum adipsin levels decreased significantly throughout normal human pregnancy. Adipsin levels in preeclamptic pregnant women in early and middle pregnancy were very similar to the ones observed in normal pregnant women. However, serum adipsin levels were significantly higher in preeclamptic women towards the end of gestation. Also, in the postpartum group, serum adipsin levels were higher than in the normal pregnant group. On the other hand, adipsin levels were negatively correlated to glucose, total cholesterol, and LDL-cholesterol.

Moreover, adipsin was positively correlated with weight and BMI during early and late pregnancy.

In this manner, adipsin, factor B, ASP, and its precursor C3 are synthesized in adipose tissue^3^. Saleh *et al.*, in a cross-sectional study, analyzed the plasma levels of ASP in pregnant women during late pregnancy (months 8–9) and in non-pregnant women controls, finding that circulating ASP levels rise significantly in late pregnancy compared to controls^37^. This increase in ASP serum levels in late pregnancy is contrary to the significant reduction in adipsin levels in pregnant women that we found in this study. In the study of Saleh *et al.* ASP serum levels were correlated positively with triglyceride levels in pregnant and non-pregnant women, while in this study no correlation was observed between triglycerides and adipsin^37^.

Sivakumar *et al.* reported the production of adipsin and ASP in placenta and adipose tissue explants. They found that the placenta produces more ASP than adipose tissue, which could explain the elevated ASP circulating levels at the end of pregnancy described previously by Saleh *et al.*^37^,^38^. It is even possible that preeclampsia could be associated with placental dysfunction, related to the changes in adipsin and ASP expression and secretion patterns in pregnant women and in the fetus^38^.

### Correlation among BMI, glucose, cholesterol, BP, and adipsin serum levels throughout pregnancy

Adipsin is an adipocytokine secreted in large amounts by adipose tissue and its levels are elevated in obese patients and positively correlated with BMI^1^,^39^. The anabolic condition of pregnant women contributes to a positive energy balance and a significant increase in the recruitment of fat mass. In spite of this, we found that adipsin levels were significantly lower throughout the entire pregnancy when compared to levels in healthy non-pregnant women. Possible explanations for these results include: the capacity of adipocytes and other tissues, such as lung and muscle, to secrete adipsin could be diminished^40^, or the degradation of serum adipsin could be higher during normal pregnancy in comparison to non-pregnant women. Although it is not possible to analyze the adipose tissues of pregnant women, it was reported that the circulating concentration of adipsin tends to correlate positively with the degree of adiposity in obese individuals^26^.

Nevertheless, circulating adipsin levels were lower during gestation; when adipsin levels were analyzed in each period of gestation, we found a positive correlation with weight and BMI in the first and last period. There are previous reports indicating that maternal adipsin levels are increased in obese pregnant women in comparison to lean pregnant women^38^. The reason for the increased levels of adipsin in obese pregnant women was attributed to the higher secretion of ASP from adipose and placental tissue^37^,^38^,^41^. Adipsin contributes to the formation of ASP, a factor involved in lipid storage in adipose tissue^29^, by increasing diacylglycerol O-acyltransferase 2 activity and reducing hormone-sensitive lipase activity^24^,^42^,^43^. We also found a significant correlation between adipsin levels and BMI after three months postpartum, suggesting an association between adipsin and adiposity after pregnancy. In another study, Saleh *et al.* showed that the BMI is not a predictor of ASP levels during pregnancy, whereas in the present study a significant positive correlation was found between BMI and adipsin levels during early and late normal pregnancy and three months after childbirth and in early pregnancy in preeclamptic women. Similarly, weight correlates with adipsin levels during the first and second trimester in normal pregnant women^44^. These results imply that the approximately 30% increase in weight gain throughout pregnancy and in fatty acid levels could not be responsible for variations in adipsin levels.

The strong inverse and significant correlation between adipsin levels compared to glucose serum levels, total cholesterol, and c-LDL during late pregnancy in preeclamptic women is important^44^. In addition, in the present study, a significant reduction in the c-HDL levels are clearly seen in early and late pregnancy in preeclamptic women when compared to non-pregnant women, a characteristic that has been previously reported^12^,^45^. In this study, during late normal pregnancy, a significant positive correlation between systolic BP and adipsin serum levels has been observed. Meanwhile in the present study, this correlation was negative in preeclamptic women. Thus, serum adipsin levels may be affected by biochemical parameters related to glucose homeostasis and cholesterol metabolism during late pregnancy in preeclamptic women.

Although, we found that adipsin levels were significantly higher in late pregnancy in preeclamptic women, at this point adipsin levels were also negatively correlated with glucose, total cholesterol, and LDL-cholesterol. This suggests that adipsin serum levels in late pregnancy in preeclamptic women could not explain the altered lipid metabolism present during this period.

### Hormonal regulation and adipsin levels through pregnancy

Our findings of lower adipsin levels through pregnancy could be explained by hormonal regulation. It has been reported in an *in vitro* study that insulin has a negative effect on adipsin mRNA levels^46^. Thus, it could be implied that a decrease in adipsin levels during pregnancy may be due in some part to the increasing levels of insulin. It has been demonstrated, that in undifferentiated adipocytes 3T3 F442A, insulin blocks adipsin secretion as a result of diminished mRNA levels. This contrasts with what is seen in pre-adipocytes, in which insulin promotes the expression of adipsin to favor the process of differentiation^47^. Additionally, studies in mice have proven that the administration of bovine placental lactogen diminishes serum adipsin levels^48^. This information correlates with the effect that lactogen has over insulin, which has been reported in *in vitro* studies where the addition of human placental lactogen and several other equivalents stimulate pancreatic β cells with an increase in insulin secretion^49^. Accordingly, adipsin synthesis and secretion might be controlled indirectly by the function of placental lactogen or several of its equivalents, which leads to a hyperinsulinemic state and a reduction of adipsin levels.

Leptin and adiponectin, two hormones that participate in the regulation of energy balance, are produced in the placenta and might contribute to the insulin and ASP resistance seen in the pregnant and fetal population. It has been proven that in the late stage of pregnancy circulating levels of leptin are significantly higher in the mother when compared to those in the fetus. On the other hand, adiponectin levels are significantly higher in the fetus in comparison to the levels of the healthy pregnant mother^50^. It is possible that the high levels of adiponectin and low levels of leptin in the fetus may contribute to the increase of ASP and insulin action on fetal tissue during the last stage of the pregnancy^38^,^51^. Although the profile of adipsin has been described in the present study, it might be relevant to determine the relationship between mother and fetal leptin levels in the end stages of pregnancy. The regulation mechanism of adipsin levels in the pregnant and fetal population is still unclear and it requires the development of studies which may unveil this phenomenon. In addition, studies have described that at the end stage of pregnancy, an increase in leptin levels and an increase of adipocytokine resistance occurs^52^,^53^. However, during the same stage of the pregnancy, we can observe a significant reduction of adiponectin levels and an elevation of insulin resistance^54^,^55^,^56^. The present study demonstrates that while leptin levels are significantly elevated with increasing gestational period in healthy pregnant women, there is no significant correlation between circulating levels of leptin and adipsin in any of the other studied periods. In addition, no significant correlation between adipsin and leptin is observed three months post - partum nor in non-pregnant healthy women during the follicular and luteal phase of the menstrual cycle.

Finally, it has been shown that in *in vitro* studies of cultured 3T3-L1 adipocytes co-cultured with J774 macrophages and treated independently with leptin, adiponectin, and TNF-α, induce the expression of ASP^57^. Thus, it is possible that leptin, adiponectin and other adipokines assist in controlling complement elements throughout gestation.

### Adipsin levels in preeclamptic women and complement

As previously described, serum adipsin levels increased significantly in the late period of gestation in preeclamptic women when compared with normal pregnant women. Additionally, a significant increase in BMI in preeclamptic women was observed. Recent studies have demonstrated that adipsin levels were significantly elevated in the urine of preeclamptic patients^22^, thus correlating with the elevation of the serum adipsin levels in late pregnancy observed in the present study^22^. Previous studies have shown that plasma levels of C3a and C5a were elevated significantly in preeclamptic patients^51^.

Buurma *et al.*, through immunohistochemistry studies, found that C4d was rarely present in placentas from healthy controls (3%), whereas it was observed in 50% of placentas obtained from preeclamptic women. In preeclamptic women, diffuse placental C4d was associated with a significantly lower gestational age at delivery. Furthermore, they found that the mRNA expression of the complement regulatory proteins, CD55 and CD59, was significantly up-regulated in placenta of preeclamptic women^13^. Lokki *et al.* also found differences between women with and without PE in the classical pathway of C activation and in the binding of protective C regulators to the placental structures, in their small cohort, partial C4A deficiency was more frequent in preeclampsia than controls^14^. In the complement pathway, C3b along with Factor B combine to form C3bB. Then, the enzyme adipsin cleaves the bound B to generate Ba and C3bBb, the active convertase that cleaves C3 to C3b and C3a. Thus, C3b can combine with factor B and start another cycle within the complement pathway. Additionally, the enzyme carboxypeptidase B (CpB) cleaves the N-terminal arginine of C3a to produce ASP^3^. Previous studies have determined the complement-activation of fragment Bb in preeclamptic and healthy pregnant women in early and late stages of pregnancy. A significant increase was observed in the levels of factor Bb in the group of women with preeclampsia than in controls^58^,^59^. Thus, the rise in Bb levels correlates with increased adipsin levels in the present study, where a statistically significant increment during the late pregnancy of preeclamptic women is observed. The present study leads us to suggest that adipsin may have an important role in the activation of complement fragments, due to their function as serine protease and its direct action on factor B, to yield the active fragment Bb, which has been associated with the pathogenesis and development of preeclampsia^58^,^59^.

Previous studies in mice have demonstrated a significant rise in the adipsin levels in aborted placenta. Adipsin is related with the activation of complement pathway, in the decidua fibroblast, which helps with the activation of the defense mechanisms of the extracellular placental tissue^60^. In humans and murines, the activation of C3 and C4 is regulated by DAF and MCP, while in mice, Crry protein participates in this regulation^8^. In endometrial samples from mouse miscarriages and human placentas, the expression of DAF, MCP, and Crry proteins has been demonstrated^60^,^61^. Recently, it has been demonstrated in CBA/J×DBA/2 model mice a significant elevation in serum and placenta adipsin levels and that these levels are related to spontaneous abortion^62^. In the present study, the rise of adipsin serum levels in late pregnancy in the preeclamptic group compared to normal pregnant women is observed. These studies, like our results, show evidence for the relationship between adipsin, increased classical pathway activation, and altered complement regulation in preeclampsia. Finally, it can be seen that the serum leptin levels are significantly elevated in each respective period in preeclamptic women when compared to levels of healthy pregnant women, but we failed to find a significant correlation between levels of leptin and adipsin in preeclamptic women.

In conclusion, this case-control study nested in a prospective cohort showed for the first time that: a) serum adipsin levels did not vary throughout the menstrual cycle of healthy non-pregnant women; b) serum adipsin levels were decreased during the entire normal pregnancy, increasing its value again at three months postpartum; c) at the early and late stages of pregnancy, circulating adipsin levels correlated positively with weight and BMI in normal pregnant women; d) serum adipsin levels increased significantly in preeclamptic pregnant women in late pregnancy when compared with normal pregnant women, and at which point serum adipsin is negatively correlated with glucose and cholesterol; e) a significant correlation was not found between leptin and adipsin during the three periods of gestation studied in healthy pregnant and preeclamptic women. Thus, it is possible that adipsin may contribute to the complex metabolic control that occurs during pregnancy.

## Methods

### Ethics statement

This study was carried out in accordance with the Declaration of Helsinki and with approval of the Ethics Committee Board of the School of Medicine of the Universidad Nacional de Colombia. The recruitment was headed by the Obstetrics & Gynecology and Physiology Department of the School of Medicine of the Universidad Nacional de Colombia between 2012 and 2014 at the Hospital de Engativa (Bogota, Colombia). Written informed consent was obtained from each participant.

### Subjects and study design

This is a case-control study nested in a longitudinal cohort of 450 normal women attending prenatal care at the Hospital de Engativa (Bogota, Colombia).

This age – matched study includes 54 normal pregnant women (control group), 18 pregnant women who developed preeclampsia without severe features (case group), 18 of the 54 normal pregnant women were followed three months postpartum. The control group involved pregnant women with normal obstetric and perinatal outcomes randomly selected from the entire cohort. To ensure that the women were representative of the general population of pregnant women of this study, subjects were selected randomly from the full cohort based on demographic data. All patients of the entire cohort who developed preeclampsia were included in the case group. Additionally, 20 healthy non-pregnant women were studied during the follicular (days 3–5) and luteal phases (days 20–22) of their menstrual cycle. The non-pregnant women who participated in the study were recruited during the same period. This group of non-pregnant women was included because in previous studies it has been shown that during the menstrual cycle some hormonal factors change over this period. Therefore a reference level of this factor during the follicular and luteal phase should exist, to be compared to levels in pregnant women^63^,^64^.

Women who initiated prenatal care between the 11^th^ and 13^th^ week of gestation, which was determined by early ultrasound and last menstrual period, were eligible to participate in this study. All of these women had a normal pregnancy, delivery at term, with no medical or obstetrical complications. They were studied during the early (12.1, range: 11.5–12.5 weeks of gestation), middle (24.3, range: 24.13–24.6 weeks of gestation), and late stages of pregnancy (34.4, range: 34.2–35.2 weeks of gestation), and part of them were followed until three months after delivery.

The diagnosis of preeclampsia was according to the evidence-based recommendations of the American College of Obstetricians and Gynecologists. Their criteria establishes the diagnosis of preeclampsia without severe features with a blood pressure ≥140/90 mmHg screened on two occasions at least 4 hours apart after a 20 week gestation period (in a woman with a previously normal blood pressure), and proteinuria (≥300 mg per 24-hour urine collection). Severe preeclampsia was established with a blood pressure ≥160/110 mmHg on two occasions at least 4 hours apart after 20 weeks of gestation in a woman with a previously normal blood pressure or with any of the following features: thrombocytopenia, renal insufficiency, impaired liver function, pulmonary edema, and cerebral or visual symptoms^65^.

The exclusion criteria at the start of this study were: history of diabetes mellitus; gestational diabetes mellitus; past or present history of thyroid disease; vascular disease; chronic hypertension; renal disease; polycystic ovary syndrome; or use of corticosteroids, β-blockers, β-agonists, and other drugs that could affect the metabolism.

### Laboratory assays

Whole blood samples were collected into 5.0-mL BD Vacutainer® serum tubes from an upper arm vein, between 7:00 and 8:00 am after overnight fasting. The blood samples were left at room temperature for 20 minutes, and then the clotted blood was centrifuged at 3500 rpm for 10 minutes at 4 °C. Serum samples were immediately transferred and stored in separate tubes at −80 °C to avoid freezing/thawing until the biochemical and hormonal analysis were performed. Serum glucose, triglycerides, total cholesterol, and high-density cholesterol (HDL) levels were measured by enzymatic methods (SPINREACT, Santa Coloma, Spain). Insulin levels were measured by a chemiluminescence assay (LIAISON Analyzer; DiaSorin S.p.A., Saluggia, Italy). Homeostasis model assessment of estimated insulin resistance (HOMA-IR) was determined using the formula described previously by Matthews *et al.*^66^: HOMA = fasting insulin (microunits per milliliter) X fasting glucose (millimoles per liter)/22.5). Serum progesterone levels were measured in healthy non-pregnant women by immunoassay (Roche Elecsys 1010 Immunoanalyzer Boulder, Colorado, USA).

### Determination of human adipsin and leptin in serum

Serum adipsin levels were quantified using a commercially available Human ELISA Kit, according to the manufacturer’s protocols (Abcam®, USA; catalog number ab99969). The intra-assay and inter-assay variation coefficients were <10% and <12%, respectively. The sensitivity of the assay was less than 4 pg/mL. Serum adipsin concentrations (ng/mL) were measured in duplicate for each participant, and the mean and the SD of the two measurements for each patient were used in the analysis. This ELISA kit shows no cross – reactivity with other cytokines tested.

Additionally, serum leptin levels were quantified using a commercially available Human ELISA Kit, according to the manufacturer’s recommendations (Invitrogen^®^, USA catalog number KAC2281). The intra-assay and inter-assay variation coefficients were <3.9 and <5.3%, respectively the sensitivity of the assay was less than <3.5 pg/mL and the leptin ELISA kit shows no cross – reactivity with other cytokines tested. Also, serum leptin concentrations (pg/mL) were analyzed in duplicate for each patient.

### Statistical analysis

Statistical tests were conducted using R software (version 3.1.1). Data with normal distribution were reported as mean +/− standard deviation (SD), while data with non-normal distribution were reported as median and interquartile range (IQR). Statistical differences between groups were tested on the anthropometric and metabolic variables using the Friedman’s test followed by the Wilcoxon signed-rank test for repeated measurements. The Wilcoxon-Mann-Whitney test was used to compare unpaired groups. The correlation between adipsin serum levels and anthropometric and metabolic variables throughout pregnancy was studied as well. Univariate correlations were assessed in the groups by Spearman’s partial correlation coefficient with adjustments for gestational age. Statistical values are presented as *p < 0.05, **p < 0.01 and ***p < 0.001. Significance was assumed at p < 0.05.

## Additional Information

**How to cite this article**: Poveda, N. E. *et al.* Serum Adipsin Levels throughout Normal Pregnancy and Preeclampsia. *Sci. Rep.* **6**, 20073; doi: 10.1038/srep20073 (2016).

## Supplementary Material

Supplementary Information

## Figures and Tables

**Table 1:** Anthropometric, clinical, and biochemical parameters and adipsin levels of normal pregnant women

Variable	Normal pregnancy (n = 54)			P-value in the Friedman’s test*	P-value in the post-hoc analyses		
	EP	MP	LP		EP Vs MP	EP Vs LP	MP Vs LP
Weight, Kg (mean ± SD)	55.6 (+/− 6.9)	60.2 (+/− 7.6)	64.3 (+/− 7.4)	0.00***	0.00***	0.00***	0.00***
BMI, Kg/m^2^ (median (IQR))	22.34 (20.46–23.7)	23.96 (22.52–25.89)	25.95 (24.4–27.48)	0.00***	0.00***	0.00***	0.00***
Gestational age, weeks (median (IQR))	12.1 (11.5–12.5)	24.3 (24.13–24.6)	34.4 (34.2–35.2)	0.00***	0.00***	0.00***	0.00***
Systolic BP, mmHg (median (IQR))	95 (90–100)	90 (86–100)	97 (90–102)	0.03*	0.12	0.38	0.04*
Diastolic BP, mmHg (median (IQR))	60 (60–63.5)	60 (58–60)	62 (60–69.5)	0.00**	0.22	0.07	0.01**
Mean BP, mmHg (mean ± SD)	72.3 (+/− 5.5)	71 (+/− 5.2)	74.2 (+/− 7.1)	0.01*	0.13	0.03*	0.01**
Glucose, mg/dL (median (IQR))	78 (73–82.75)	72 (69–77.75)	73.5 (69–77.75)	0.00***	0.00***	0.00**	0.72
Insulin, ng/mL (median (IQR))	9.5 (5.75–12)	11.2 (8.3–14.5)	12.3 (8.05–17.52)	0.00**	0.02*	0.00***	0.03*
HOMA-IR (median (IQR))	1.695 (1.129–2.217)	1.9 (1.452–2.631)	2.255 (1.575–3.139)	0.02*	0.14	0.00***	0.01*
Total cholesterol, mg/dL (mean ± SD)	166.3 (+/− 31.3)	218.7 (+/− 37.5)	247.9 (+/− 48.4)	0.00***	0.00***	0.00***	0.00***
HDL-cholesterol, mg/dL (mean ± SD)	57.4 (+/− 10.8)	69.3 (+/− 11.5)	67.2 (+/− 11.4)	0.00***	0.00***	0.00***	0.13
LDL-cholesterol, mg/dL (mean ± SD)	119.3 (+/− 33.3)	146.7 (+/− 43.2)	157 (+/− 41.5)	0.00***	0.00***	0.00***	0.01**
VLDL-cholesterol, mg/dL (median (IQR))	21.58 (17.54–25.98)	35.46 (28.06–43.24)	46.54 (40.37–56.14)	0.00***	0.00***	0.00***	0.00***
Triglycerides, mg/dL (median (IQR))	107.9 (87.7–129.9)	177.3 (140.3–216.2)	223.6 (201.1–282.4)	0.00***	0.00***	0.00***	0.00***
Adipsin, ng/mL (mean ± SD)	4381 (+/− 663.4)	4083 (+/− 655.1)	4314.5 (+/− 665)	0.04*	0.01**	0.68	0.02*
Leptin, pg/mL (mean ± SD)	23461.12 (+/− 9011.357)	34112.42 (+/− 20165.563)	48144.76 (+/− 31264.723)	0.00***	0.00***	0.00***	0.00***

Non-normally distributed data are listed as median (IQR). Normally distributed data are listed as mean ± SD. EP = early pregnancy, MP = middle pregnancy, LP = late pregnancy, PP = three months postpartum. HOMA (Homeostasis Model Assessment). *p-value < 0.05, **p-value < 0.01, ***p-value < 0.001.

**Table 2:** Anthropometric, clinical and biochemical parameters and adipsin levels of preeclamptic women

Variable	Preeclamptic women (n = 18)			P-value in the Friedman’s test*	P-value in the post-hoc analyses		
	EP	MP	LP		EP Vs MP	EP Vs LP	MP Vs LP
Weight, Kg (mean ± SD)	58.5 (+/− 6.7)	65.6 (+/− 7.7)	73.2 (+/− 8.5)	0.00***	0.00***	0.00***	0.00***
BMI, Kg/m^2^ (median (IQR))	23.14 (21.63–24.75)	26.09 (24.06–28.04)	29.74 (27.36–30.59)	0.00***	0.00***	0.00***	0.00***
Gestational age, weeks (median (IQR))	12.25 (11.6–12.58)	24.3 (24.1–24.5)	35.2 (34.28–35.58)	0.00***	0.00***	0.00***	0.00***
Systolic BP, mmHg (median (IQR))	108 (98.5–110)	100 (100–110)	104 (100–110)	0.18	—	—	—
Diastolic BP, mmHg (median (IQR))	65 (60–70)	63 (60–68)	60 (60–70)	0.82	—	—	—
Mean BP, mmHg (mean ± SD)	78.2 (+/− 7.5)	77.6 (+/− 5.6)	77 (+/− 4)	0.56	—	—	—
Glucose, mg/dL(median (IQR))	80.5 (74.65–84)	78.5 (70–81)	70.6 (69–75)	0.01**	0.05*	0.00**	0.08
Insulin, ng/mL (median (IQR))	11.5 (10.3–13.3)	14.75 (11.05–18.32)	13.5 (11.42–17.82)	0.22	—	—	—
HOMA-IR (median (IQR))	2.351 (1.889–2.508)	2.946 (2.195–3.649)	2.451 (2.116–3.348)	0.27	—	—	—
Total cholesterol, mg/dL (mean ± SD)	182.2 (+/− 21.3)	213 (+/− 33.6)	237.7 (+/− 50.8)	0.00***	0.00***	0.00***	0.06
HDL-cholesterol, mg/dL (mean ± SD)	50.1 (+/− 10.6)	62.5 (+/− 14.8)	55.4 (+/− 7.2)	0.07	—	—	—
LDL-cholesterol, mg/dL (mean ± SD)	124.2 (+/− 40.5)	145.1 (+/− 43.6)	162 (+/− 70.7)	0.01*	0.01**	0.02*	0.21
VLDL-cholesterol, mg/dL (median (IQR))	21.05 (16.23–26.48)	32.8 (27.97–36.45)	46.86 (32.12–62.19)	0.00***	0.00***	0.00***	0.01**
Triglycerides, mg/dL (median (IQR))	105.2 (81.15–132.4)	164 (139.8–182.2)	243.7 (183.3–311)	0.00***	0.00***	0.00***	0.00***
Adipsin, ng/mL (mean ± SD)	4764.4 (+/− 1414.4)	3927.4 (+/− 674.8)	5932.1 (+/− 2036.2)	0.00**	0.01**	0.18	0.00***
Leptin, pg/mL (mean ± SD)	33734.14 (+/−11928.92)	61060.55 (+/−23970.139)	89780.93 (+/−41171.870)	0.00***	0.00***	0.00***	0.027*

Non-normally distributed data are listed as median (IQR). Normally distributed data are listed as mean ± SD. EP = early pregnancy, MP = middle pregnancy, LP = late pregnancy, PP = three months postpartum. HOMA (Homeostasis Model Assessment).
