# Supplementary material for: Serum Adipsin Levels throughout Normal Pregnancy and Preeclampsia
Source: Sci Rep. 2016 Feb 1;6:20073. doi: 10.1038/srep20073 (PMC4735521; doi:10.1038/srep20073)
Supplement: Supplementary Information [file srep20073-s1.pdf]

Supplementary Information

Serum Adipsin Levels throughout Normal Pregnancy and Preeclampsia

Natalia E. Poveda, María F. Garcés, Carlos E. Ruiz-Linares, Diana Varón, Sergio Valderrama, Elizabeth Sanchez, Adriana Castiblanco-Cortes, Yessica Agudelo-Zapata, Héctor Fabio Sandoval-Alzate, Luis G. Leal, Edith Ángel-Müller, Ariel I. Ruíz-Parra, Angélica M. González-Clavijo, Carlos Diéguez, Rubén Nogueiras and Jorge E. Caminos

Supplemental Table 1. Anthropometric, clinical and biochemical parameters and hormonal levels of healthy eumenorrheic women

Variable	Eumenorrheic Women		
	Follicular phase (n=20)	Luteal phase (n=20)	p-value
Progesterone, ng/mL (mean ± SD)	0.4952 (0.3284-0.6569)	6.428 (3.785-13.45)	0.00***
Adipsin, ng/mL (mean ± SD)	5357.9 (+/- 714.4)	5393.9 (+/- 921.5)	0.89
Leptin, pg/mL (mean ± SD)	16316.0(+/- 2304.729)	23018.85 (+/- 4711.553)	0.00***
Age, years, (median (IQR))	20 (19-23.25)		
Height, meters (mean ± SD)	1.6 (+/- 0.1)		
Weight, Kg (mean ± SD)	55.5 (+/- 6.4)		
BMI, Kg/m^2 (mean ± SD)	21.6 (+/- 2.2)		
Systolic BP, mmHg (mean ± SD)	108.3 (+/- 8.3)		
Diastolic BP, mmHg (mean ± SD)	71.2 (+/- 7.2)		
Mean BP, mmHg (mean ± SD)	83.6 (+/- 6.2)		
Insulin, ng/mL (mean ± SD)	9.1 (+/- 5)		
Total cholesterol, mg/dL (mean ± SD)	157.9 (+/- 24.8)		
HDL-cholesterol, mg/dL (mean ± SD)	52.1 (+/- 8.4)		
Triglycerides, mg/dL (mean ± SD)	75.7 (+/- 19.3)		
VLDL-cholesterol, mg/dL (mean ± SD)	15 (+/- 3.9)		
LDL-cholesterol, mg/dL (mean ± SD)	109 (+/- 21.9)		
PCR, mg/L (mean ± SD)	1.5 (+/- 0.8)		
Glucose, mg/dL (mean ± SD)	84.9 (+/- 7.3)		
HOMA-IR (mean ± SD)	1.9 (+/- 1.1)		

Non-normally distributed data are listed as median (IQR). Normally distributed data are listed as mean ± SD. HOMA (Homeostasis Model Assessment). ***p-value < 0.001.

Supplementary Table 2. A comparison of anthropometric, clinical and biochemical parameters and adipsin levels between healthy women across pregnancy and three months postpartum and preeclamptic women

Variable	EP			MP			LP			PP	
	Healthy pregnancy (n=54)	Preeclamptic pregnancy (n=18)	p-value	Healthy pregnancy (n=54)	Preeclamptic women (n=18)	p-value	Healthy pregnancy (n=54)	Preeclamptic pregnancy (n=18)	p-value	Healthy pregnancy (n=18)	p-value*
Age, years, median (IQR)	23 (19-30)	19.5 (18.25-25.5)	0.14	23 (19-30)	19.5 (18.25-25.5)	0.14	23 (19-30)	19.5 (18.25-25.5)	0.14	23 (19-24.75)	0.49
Weight, Kg (mean ± SD)	55.6 (+/- 6.9)	58.5 (+/- 6.7)	0.14	60.2 (+/- 7.6)	65.6 (+/- 7.7)	0.01*	64.3 (+/- 7.4)	73.2 (+/- 8.5)	0.00 ***	57 (+/- 6.4)	0.00 ***
Height, meters , (median (IQR))	1.58 (1.542-1.598)	1.585 (1.53-1.618)	0.75	1.58 (1.542-1.598)	1.585 (1.53-1.618)	0.75	1.58 (1.542-1.598)	1.585 (1.53-1.618)	0.75	1.558 (1.542-1.58)	0.57
BMI, Kg/m^2 (median (IQR))	22.34 (20.46-23.7)	23.14 (21.63-24.75)	0.12	23.96 (22.52-25.89)	26.09 (24.06-28.04)	0.01*	25.95 (24.4-27.48)	29.74 (27.36-30.59)	0.00 ***	23.63 (21.54-25.05)	0.00 ***
Gestational age, weeks (median (IQR))	12.1 (11.5-12.5)	12.25 (11.6-12.58)	0.75	24.3 (24.13-24.6)	24.3 (24.1-24.5)	0.87	34.4 (34.2-35.2)	35.2 (34.28-35.58)	0.09	-	-
Systolic BP, mmHg (median (IQR))	95 (90-100)	108 (98.5-110)	0.00 ***	90 (86-100)	100 (100-110)	0.00 ***	97 (90-102)	104 (100-110)	0.00 **	102 (90-109)	0.30
Diastolic BP, mmHg (median (IQR))	60 (60-63.5)	65 (60-70)	0.10	60 (58-60)	63 (60-68)	0.01 **	62 (60-69.5)	60 (60-70)	0.81	62 (60-64)	0.86
Mean BP, mmHg (mean ± SD)	72.3 (+/- 5.5)	78.2 (+/- 7.5)	0.01 **	71 (+/- 5.2)	77.6 (+/- 5.6)	0.00 ***	74.2 (+/- 7.1)	77 (+/- 4)	0.05	75 (+/- 6.8)	0.68
Glucose, mg/dL (median (IQR))	78 (73-82.75)	80.5 (74.65-84)	0.12	72 (69-77.75)	78.5 (70-81)	0.03*	73.5 (69-77.75)	70.6 (69-75)	0.54	79 (77-83)	0.00 ***
Insulin, ng/mL (median (IQR))	9.5 (5.75-12)	11.5 (10.3-13.3)	0.04 *	11.2 (8.3-14.5)	14.75 (11.05-18.32)	0.00 **	12.3 (8.05-17.52)	13.5 (11.42-17.82)	0.48	5.8 (3.9-8.75)	0.00 ***

HOMA-IR (median (IQR))	1.695 (1.129-2.217)	2.351 (1.889-2.508)	0.04 *	1.9 (1.452-2.631)	2.946 (2.195-3.649)	0.00 ***	2.255 (1.575-3.139)	2.451 (2.116-3.348)	0.61	1.146 (0.7481-1.75)	0.00 ***
Total cholesterol, mg/dL (mean ± SD)	166.3 (+/- 31.3)	182.2 (+/- 21.3)	0.03 *	218.7 (+/- 37.5)	213 (+/- 33.6)	0.55	247.9 (+/- 48.4)	237.7 (+/- 50.8)	0.46	158.5 (+/- 31.3)	0.00 ***
HDL-cholesterol, mg/dL (mean ± SD)	57.4 (+/- 10.8)	50.1 (+/- 10.6)	0.02 *	69.3 (+/- 11.5)	62.5 (+/- 14.8)	0.09	67.2 (+/- 11.4)	55.4 (+/- 7.2)	0.00 ***	48.2 (+/- 7.4)	0.00 ***
LDL-cholesterol, mg/dL (mean ± SD)	119.3 (+/- 33.3)	124.2 (+/- 40.5)	0.65	146.7 (+/- 43.2)	145.1 (+/- 43.6)	0.90	157 (+/- 41.5)	162 (+/- 70.7)	0.78	90.3 (+/- 24.1)	0.00 ***
VLDL-cholesterol, mg/dL (median (IQR))	21.58 (17.54-25.98)	21.05 (16.23-26.48)	0.77	35.46 (28.06-43.24)	32.8 (27.97-36.45)	0.13	46.54 (40.37-56.14)	46.86 (32.12-62.19)	0.76	13 (10.22-19)	0.00 ***
Triglycerides, mg/dL (median (IQR))	107.9 (87.7-129.9)	105.2 (81.15-132.4)	0.77	177.3 (140.3-216.2)	164 (139.8-182.2)	0.13	223.6 (201.1-282.4)	243.7 (183.3-311)	0.40	66 (51.5-96)	0.00 ***
Adipsin, ng/mL (mean ± SD)	4381 (+/- 663.4)	4764.4 (+/- 1414.4)	0.28	4083 (+/- 655.1)	3927.4 (+/- 674.8)	0.40	4314.5 (+/- 665)	5932.1 (+/- 2036.2)	0.00 **	6401.7 (+/- 1148.8)	0.00 ***
Leptin pg/mL (mean ± SD)	23461.12 (+/- 9011.40)	33734.14(+/- 11928.92)	0.00 ***	34112.42 (+/- 20165.60)	61060.55 (+/- 23970.14)	0.00 ***	48144.76 (+/- 31264.72)	89780.93 (+/- 41171.88)	0.00 ***	17028.81 (+/- 3935.63)	0.00 ***

Non-normally distributed data are listed as median (IQR). Normally distributed data are listed as mean ± SD. EP=early pregnancy, MP=middle pregnancy, LP=late pregnancy, PP=three months postpartum. HOMA (Homeostasis Model Assessment). *Test of mean differences between normal LP and normal PP groups. * P-value < 0.05, ** p-value < 0.01, ***p-value < 0.001.

Supplemental Table 3. Univariate partial correlations between Adipsin and leptin, anthropometric/biochemical parameters.

Variable	Eumenorrheic women (n=20)		EP				MP				LP				Healthy pregnant postpartum (n=18)	
			Healthy pregnancy (n=54)		Preeclamptic pregnancy (n=18)		Healthy pregnancy (n=54)		Preeclamptic pregnancy (n=18)		Healthy pregnancy (n=54)		Preeclamptic pregnancy (n=18)			
	Correlation	p-value	Correlation	p-value	Correlation	p-value	Correlation	p-value	Correlation	p-value	Correlation	p-value	Correlation	p-value	Correlation	p-value
Weight, Kg	-0.08	0.63	0.49	0.00 ***	0.32	0.32	0.27	0.05	0.28	0.33	0.38	0.01 **	0.02	0.95	0.09	0.73
Height, meters	-0.28	0.08	0.31	0.02 *	-0.42	0.17	0.21	0.15	-0.12	0.69	0.01	0.97	-0.09	0.80	0.05	0.83
BMI, Kg/m^2	0.03	0.84	0.34	0.01 *	0.87	0.00 ***	0.20	0.17	0.37	0.19	0.39	0.01 **	0.09	0.80	0.51	0.03 *
Systolic BP, mmHg	-0.07	0.68	0.19	0.17	-0.33	0.29	-0.01	0.95	0.15	0.62	0.41	0.00 **	-0.77	0.00 ***	0.04	0.87
Diastolic BP, mmHg	-0.50	0.00 **	0.12	0.42	-0.11	0.75	-0.01	0.95	-0.45	0.09	0.22	0.14	0.53	0.08	0.55	0.02 *
Mean BP, mmHg	-0.32	0.05	0.21	0.15	-0.20	0.54	-0.04	0.81	-0.35	0.21	0.28	0.05	-0.32	0.34	0.45	0.07
Glucose, mg/dL	0.04	0.79	0.07	0.60	0.16	0.63	0.27	0.06	0.16	0.59	0.07	0.62	-0.77	0.00 ***	0.34	0.18
Insulin, ng/mL	-0.02	0.89	0.21	0.14	0.14	0.67	0.02	0.90	-0.12	0.70	0.02	0.90	0.52	0.09	0.15	0.54
HOMA-IR	0.02	0.90	0.20	0.15	0.18	0.58	0.04	0.77	-0.11	0.70	0.02	0.87	0.35	0.29	0.17	0.49
Total cholesterol, mg/dL	0.12	0.45	-0.23	0.10	0.00	0.99	0.16	0.27	-0.03	0.93	0.14	0.35	-0.76	0.00 **	0.05	0.85
HDL-cholesterol, mg/dL	-0.29	0.07	-0.07	0.61	0.57	0.04 *	0.12	0.42	0.26	0.37	-0.03	0.82	0.32	0.35	0.06	0.82
LDL-cholesterol, mg/dL	0.23	0.17	-0.22	0.12	0.02	0.95	-0.05	0.75	0.30	0.30	0.15	0.31	-0.92	0.00 ***	-0.16	0.53
Triglycerides, mg/dL	0.12	0.48	-0.11	0.42	-0.57	0.04 *	0.27	0.06	0.10	0.73	0.01	0.96	-0.09	0.80	-0.18	0.48
Leptin, pg/mL			-0.03	0.8	0.1	0.67	-0.17	0.21	0.27	0.30	0.17	0.20	0.39	.080	0.35	0.12
Follicular	-0.16	0.48														

Luteal	-0.09	0.71

EP=early pregnancy, MP=middle pregnancy, LP=late pregnancy, PP=three months postpartum. HOMA (Homeostasis Model Assessment). * P-value < 0.05, ** p-value < 0.01, ***p-value < 0.001.

Supplementary Figure 1 (A-G). Correlations between adipsin levels and anthropometric, clinical, and biochemical features in preeclamptic women

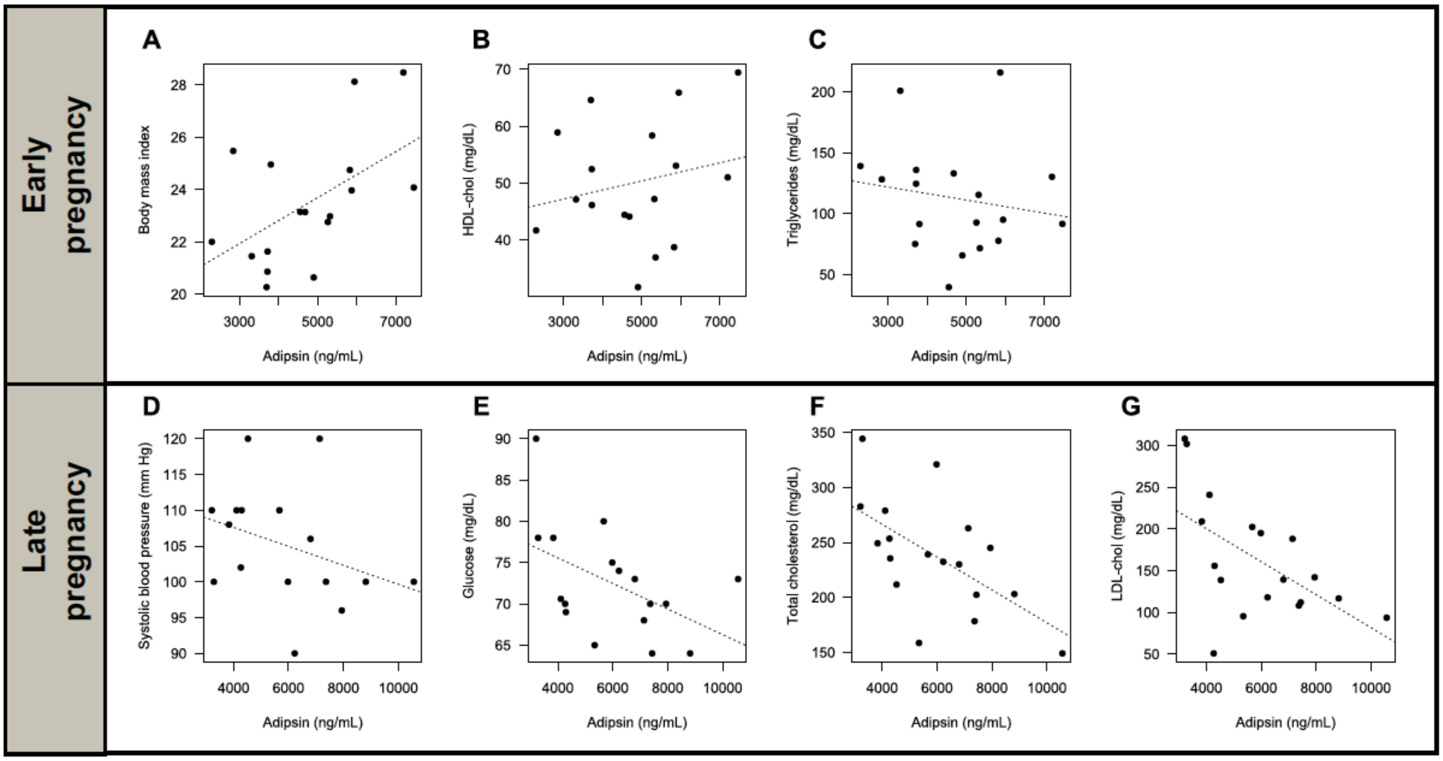

Supplementary Figure 1. Scatterplots of anthropometric/biochemical variables correlated with adipsin in preeclamptic women. (A-B) Positive correlations between serum adipsin levels and BMI and HDL-cholesterol levels in early pregnancy. (C) Negative correlation between adipsin levels and triglycerides in early pregnancy. (D-G) Negative correlations between adipsin levels and systolic BP, glucose levels, total cholesterol, and LDL-cholesterol levels in late pregnancy.
